# Leaflet anatomy verifies relationships within *Syagrus* (Arecaceae) and aids in identification

**DOI:** 10.3897/phytokeys.26.5436

**Published:** 2013-09-30

**Authors:** Larry R. Noblick

**Affiliations:** 1 Montgomery Botanical Center, 11901 Old Cutler Road, Miami, Florida 33156, USA

**Keywords:** leaflet anatomy, identification, Arecaceae, *Syagrus*, acaulescent

## Abstract

The current investigation was carried out to examine how palm anatomy may coincide with the current molecular analysis including the three recognized clades of *Syagrus* Mart. and to justify the splitting of acaulescent *Syagrus* species (e.g. *Syagrus petraea* (Mart.) Becc.) into several species. Free-hand cross-sections of leaflets were made and the comparison of these verifies the relationships suggested by the molecular data. Free-hand leaflet sections were also found to be useful in the identification of otherwise difficult-to-identify acaulescent *Syagrus* species. The result and conclusion is that anatomical data is valuable in helping to verify molecular data and that splitting the acaulescent species of *Syagrus* is justified by the differences discovered in their field habit and anatomy. These differences were used to produce an identification key that is based on the anatomy.

## Introduction

*Syagrus* is part of the largest subfamily of palms, Arecoideae (Dransfield et al. 2005, 2008; Gunn 2004). Recent construction of a supertree using a supermatrix consisting of DNA sequence data, plastid restriction fragment length polymorphism data and morphological data further supports that *Syagrus* belongs to the Cocoseae (Baker et al. 2009). Within the Arecoideae there is strong support via the phylogenetic analyses of the low copy genes PRK and RPB2 that it belongs to the RRC (Roystoneeae, Reinhardtieae, Cocoseae) clade and specifically to the Cocoseae and Attaleinae (Baker et al. 2011). Attaleinae include genera like *Allagoptera*, *Attalea*, *Beccariophoenix*, *Butia*, *Cocos*, *Jubaea*, *Jubaeopsis*, *Lytocaryum*, *Parjubaea*, *Syagrus*, and *Voanioala* or in other words all of the non-spiny palms with small hard coconut-like fruits.

Palm leaflet anatomy has been useful in identification and has been used to suggest systematic relationships. Tomlinson (1961) examined and described the leaflet anatomy of some 250 species of palms in 137 genera and suggested some systematic relationships among genera. Horn et al. (2009) took it a step farther and mapped out the lamina anatomy using the data matrix for the palm family resulting from a phylogenetic analysis by Asmussen et al. (2006) in order to understand the lamina anatomy evolution. Tomlinson’s “brief survey” (Tomlinson 1961) inspired Glassman (1972, 1987) to examine *Syagrus* and its closely related genera in greater detail. Glassman (1972) emphasized that his survey of the genus was “based on mostly one collection for each taxon.” However by the time he completed his revision (Glassman 1987), slides of two or more specimens were made for most taxa. His key was written as a convenient tool for identification and was not intended to show close relationships. Meerow et al. (2009) showed how leaflet anatomy further supported the molecular relationships between *Allagoptera*, *Parajubaea* and *Polyandrococos* (now synonym of *Allagoptera*). Tomlinson et al. (2011) expanded his original work and presented information on 183 palm genera (out of 185 now recognized) and suggested relationships based on anatomy and the use of modern phylogenetic approaches.

Glassman (1972) emphasized the following anatomical characters: (1) nature of adaxial and abaxial surfaces (straight, wavy or furrowed), (2) number of cell layers comprising both adaxial and abaxial hypodermis, (3) relative frequency and location of larger veins and whether they bulge at the laminar surfaces, (4) frequency and location of intermediate and minor veins (whether they are abaxial, in the middle or both) and whether they are attached to nonvascular fibers (5) relative abundance, shape and location (adaxial and abaxial, or adaxial only) of clusters of nonvascular fibers (N.V.F.), called fiber bundles by Tomlinson et al. (2011), (6) relative frequency and location of stomata (abaxial surface only or both leaf surfaces) and whether they are sunken or not, (7) size of midrib and its shape in transection (truncate, rounded or pointed), (8) size and shape of expansion cell tissue (E.C.T.), (9) relative frequency of dark staining bodies (probably groups of cells containing tannins) in mesophyll and midrib, (10) number and relative size of fiber clusters and veins at extremities of pinna.

Tomlinson et al. (2011) suggested that the following anatomical features vary in *Syagrus*: (1) abundance and distribution of trichomes; (2) adaxial epidermal cells varying from rectangular, with distinctly sinuous anticlinal walls to rhombohedral, obviously extended cells with straight or at most undulate walls; (3) the thickness of the outer epidermal wall, varying from very thick with the cell lumen scarcely one tenth its depth, to thinner walls only 2-3 times thicker than the remaining walls, the cell lumen large; (4) the abundance and location of adaxial non-vascular fibers, varying from an almost continuous layer within the hypodermis to few fibers; (5) the extent and location of abaxial non-vascular fibers; (6) the extent to which the minor abaxial veins are in contact with the abaxial hypodermis; (7) the degree to which the inner sheath of major veins develop fibrous extensions to the upper surface layers.

**Figure 1.**
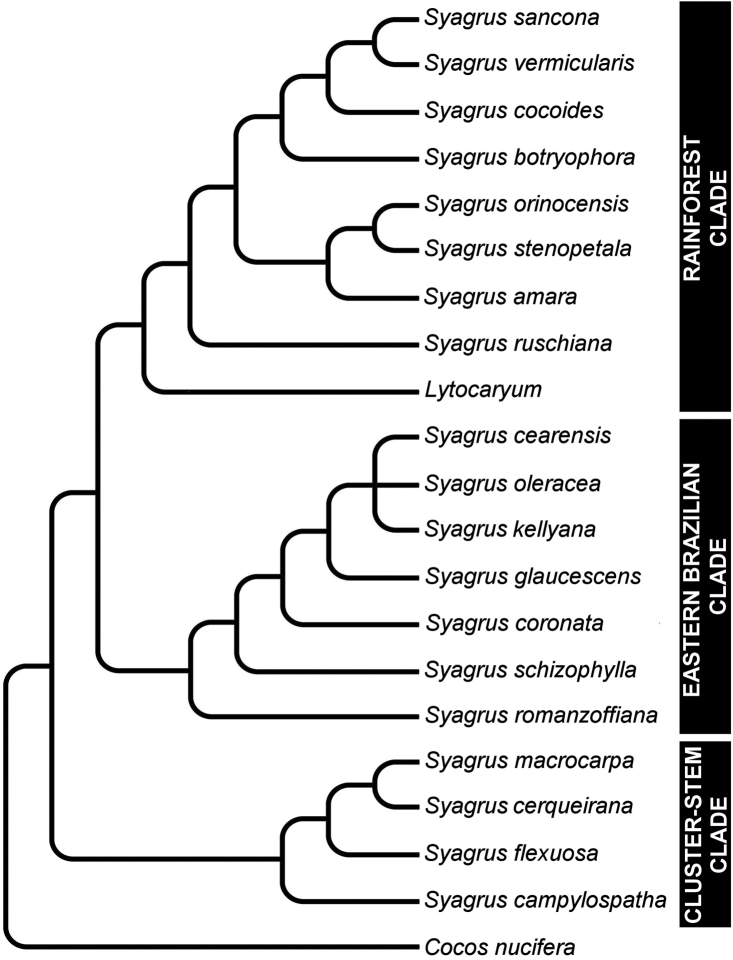
Cladogram showing major relationships in *Syagrus* (adapted from Meerow et al. 2009). Three major clades of *Syagrus* have been identified through analysis of low copy nuclear genes: the Rain Forest Clade, the Eastern Brazilian Clade, and the Cluster-stem Clade.

The first part of this paper investigates leaflet anatomy to see how it coincides and possibly even verifies the relationships supported by the molecular analysis of Meerow et al. (2009). Leaflet anatomy was useful in producing valuable anatomical characters for a phylogenetic analysis of the Attalinae palms based purely on morphological and anatomical characters (Noblick et al. 2013). Some of these anatomical characters were found to coincide with the molecular analysis of the Attaleinae (Meerow et al. 2009) as previously mentioned above. Using seven low copy nuclear WRKY genes, Meerow et al. (2009) inferred that the coconut (*Cocos nucifera*) was sister to new world *Syagrus* species and that the genus *Syagrus* was composed of at least three distinct clades: Rain Forest, Eastern Brazilian and Cluster-stem (Fig. 1). The three clades were examined anatomically to see if their anatomy supported these relationships.

The second part of this paper deals with the problem of identifying the “acaulescent” species of *Syagrus*. Most of these palms grow in Brazilian savannas (*cerrados*) and high altitude rocky fields (*campo rupestre*). Many species of *Syagrus* are described as acaulescent and Tomlinson et al. (2011) admitted that based on the low number of collections, especially of *Syagrus*, there was a great need for more detailed studies within this group. In fact, about 25 out the 54 currently recognized species of *Syagrus* are acaulescent or short stemmed (Table 1) and their identification remains challenging with many having formerly been dismissed as *Syagrus petraea* (Noblick and Lorenzi 2010). Palms that do not have visible above ground stems are often referred to as acaulescent meaning “without a stem.” In fact all palms have a stem, whether it is suberect, short-stemmed with the crown remaining at ground level, persistent juvenile which remains at ground level or rhizomatous stems that remain horizontal at ground level (Tomlinson et al. 2011). *Syagrus lilliputiana* (Barb. Rodr.) Becc., *Syagrus itacambirana* Noblick & Lorenzi, *Syagrus pleiocladoides* Noblick & Lorenziand *Syagrus procumbens* Noblick & Lorenziare suberect and short-stemmed. *Syagrus microphylla* Burret, *Syagrus vagans* (Bondar) A.Hawkes, *Syagrus werdermanii* Burret and *Syagrus duartei* Glassman usually have rhizomatous stems that remain horizontal at ground level and which are not always apparent. *Syagrus pleioclada* Burret usually has a very short, vertical aboveground stem. Over half of the acaulescent *Syagrus* species have similar looking simple spicate inflorescences (an unbranched inflorescence) and similar looking flowers and fruits. Many herbarium specimens of these acaulescent *Syagrus* look alike, but in the field they display characters that are not well preserved on herbarium sheets or that are not reported on the labels (Table 1), like deflexed pinnae (leaflets that are bent or turned abruptly or sharply downwards) or petioles that bend strongly downwards causing the leaf to lie parallel or flat on the ground (prostrated) as opposed to having leaves that are strongly ascending with straight petioles. The second part of this paper will therefore confirm, add to and hopefully correct some of the work already started by Glassman (1972, 1987). Not counting the two acaulescent *Butia* that Glassman (1987) included in the *Syagrus* portion of the leaflet anatomical key for his revision, Glassman only includes about half or 12 of the 26 currently recognized acaulescent *Syagrus* taxa. In addition, he misidentified some of the specimens used in that key. For his anatomical studies, he sometimes neglected to use specimens collected in the vicinity of their holotypes. *Syagrus petraea* (Fig. 6M) whose Bolivian holotype is now believed to be a local endemic is just such an example. His anatomical leaflet drawing of Glaziou 22254 (Glassman 1987) from central Brazil in the state of Goiás matches *Syagrus glazioviana* (Dammer) Becc. anatomy (Fig. 7E) more than it does the Bolivian *Syagrus petraea* (Fig. 6M). Also instead of Glassman’s simple drawings of the middle portion of the lamina, I believe the images of leaflet marginal cross-sections to be more useful and more informative. Therefore, the second intent of this paper is to facilitate the identification of these difficult acaulescent *Syagrus* species by (1) making use of poorly recorded field characters (Table 1) that are not easily interpreted from a flattened dried specimen and (2) their leaflet anatomy (Table 2) making use of digital images of the cross-sections of leaflet margins.

**Table 1.** Visible morphological field characters of “acaulescent” *Syagrus* species. x = normally present and s = sometimes present.

#	Name	Petiole deflexed	Pinnae deflexed	Pinnae regular	Pinnae clustered	Pinnae silvery blue	Pinnae whitish/silver beneath	Inflorescence a spike	Inflorescence branched
1	*Syagrus allagopteroides* Noblick & Lorenzi				x			x	
2	*Syagrus angustifolia* Noblick & Lorenzi				x			x	x
3	*Syagrus caerulescens* Noblick & Lorenzi	x				x	x	x	
4	*Syagrus campylospatha* (Barb.Rodr.) Becc.				x				x
5	*Syagrus cerqueirana* Noblick & Lorenzi				x				x
6	*Syagrus duartei* Glassman				x				x
7	*Syagrus evansiana* Noblick	x			x		x	x	
8	*Syagrus glazioviana* (Dammer) Becc.			s	x			x	
9	*Syagrus gouveiana* Noblick & Lorenzi				x			x	
10	*Syagrus graminifolia* (Drude) Becc.			s	x	x			x
11	*Syagrus graminifolia* var. *glazioviana* (Dammer) Becc.			s	x				x
12	*Syagrus harleyi* Glassman		s	x					x
13	*Syagrus itacambirana* Noblick & Lorenzi	s			x				x
14	*Syagrus lilliputiana* (Barb.Rodr.) Becc.	x			x				x
15	*Syagrus loefgrenii* Glassman				x		x	x	x
16	*Syagrus longipedunculata* Noblick & Lorenzi	x			x			x	
17	*Syagrus mendanhensis* Glassman				x				x
18	*Syagrus microphylla* Burret	x			x	x			x
19	*Syagrus minor* Noblick & Lorenzi	x		x	x			x	
20	*Syagrus petraea* (Mart.) Becc.				x		x	x	
21	*Syagrus pleioclada* Burret		x		x				x
21	*Syagrus pleiocladoides* Noblick & Lorenzi		x	x				x	
23	*Syagrus procumbens* Noblick & Lorenzi	x			x			x	
24	*Syagrus rupicola* Noblick & Lorenzi				x	x		x	x
25	*Syagrus vagans* (Bondar) A. Hawkes			x					x
26	*Syagrus werdermannii* Burret				x				x

**Table 2.** List of anatomical descriptors or characters for each of the species of acaulescent *Syagrus*. X = present, S = sometimes.

Descriptors or Characters	*Syagrus campylospatha*	*Syagrus harleyi*	*Syagrus cerqueirana*	*Syagrus allagopteroides*	*Syagrus lilliputiana*	*Syagrus minor*	*Syagrus loefgrenii*	*Syagrus longipedunculata*	*Syagrus angustifolia*	*Syagrus itacambirana*	*Syagrus procumbens* ‘*emasensis*’	*Syagrus procumbens*	*Syagrus petraea*	*Syagrus gouveiana*	*Syagrus duartei*	*Syagrus caerulescens*	*Syagrus rupicola*	*Syagrus evansiana*	*Syagrus glazioviana*	*Syagrus microphylla*	*Syagrus vagans*	*Syagrus werdermannii*	*Syagrus pleiocladoides*	*Syagrus pleioclada*	*Syagrus mendanhensis*	*Syagrus graminifolia*	*Syagrus graminifolia* var. *glazioviana*
**Major veins**																											
Huge major vein adjacent to or nearly adjacent to leaflet margin											X																
Normal-sized major vein near the margin but not adjacent to it (separated by a fiber bundle adaxially and/or a minor vein abaxially.								X				X													X		
Major vein usually unattached separated from the abaxial and adaxial hypodermis by another cell layer or more				X				X			?				X			X				X	X				
Major veins attached to the adaxial hypodermis but separated from the abaxial hypodermis by an additional cell layer or layers		X	X		X	X	X	X	X	X		X	X	X	X	X	X	X	X	X	X			X	X	X	X
Major veins mostly attached to the adaxial surface often by a short or long fibrous sheath extension (girder)												X		X	X	X	X	X	X		X	X					
Major veins are usually attached to both the adaxial and abaxial hypdermis.	X																					X					
**Intermediate veins**																											
Intermediate veins unattached												X			X		X	X	X				X				
Most intermediate veins attached adaxially by a fibrous sheath extension		X	X	X		X			X	X			X	X						X	X	X	X			X	X
Intermediate veins attached to both surfaces by a fibrous sheath extension	X																										
**Minor veins**																											
Minor veins on both surfaces often nearly pairing up acoss from each other			X	X	X	X					S																
Minor veins near the middle of the mesophyll or slightly below it (closer to the middle than to the abaxial)								X			X	X		S		S		X	X	X	X		X				
Many minor veins adjacent to the abaxial surface or in the lower third of the lamina (closer to the abaxial surface than to the middle)		X		X			X		X	X			X	X	X	X	X	X	S	X		X		X	X	X	X
**Veins with exaggerated fiberous sheath**																											
Vein with large exaggerated fibrous sheath running along the margin		S	S	X	X	X	X	X	X	X																	
One minor vein with exaggerated fibrous sheath running along the margin	X	X	S					X			X	S		X	X			X				S		S		X	X
Two or more minor veins with exaggerated fibrous sheath running along the margin												X				X	X	S	X	X		X		X			
**Fiber bundles**																											
Major fiber bundle running along or very near the leaflet margin															X	S	X	S									
First or second fiber bundle on adaxial surface larger than rest												X	X	X	X	X	S	X	X	X	X	X		S			
Most large adaxial fiber bundles reach ca. 1/3 to 1/2 across the mesophyll				X					X	X	X	X		X	X	X	X	X	X	X	X	X		X	X	X	X
Most small adaxial fiber bundles reach 1/5 to 1/4 across the mesophyll	X	X	X		X	F	X	X					X										X				
Adaxial fiber bundles mostly long and skinny	X		X	X			X		X	X		X	X								X	X				X	X
Adaxial fiber bundles mostly long and thick											X			X	X	X	X	X	X	X					X		
Adaxial fiber bundles mostly short and thick		X			X	X		X															X	X	S		
Minor fiber bundles present among or between larger adaxial fiber bundles and veins	S	X	X		X	X	X		X	X		X	X		S	X	X	X	X	X			X	X		X	
Minor fibers or fiber bundles scattered in the mesophyll																				X	X	X					
Minor fiber bundles along the abaxial surface absent			X	X		X	X			X			X	X	X										X	S	X
Few minor fiber bundles along the abaxial surface	X		S													X	X							X		S	
Many minor fiber bundles adjacent to the abaxial surface		X	S					X			X	X					S	X	X	X	X	X	X				
Abaxial minor fiber bundles occasionally alternating with abaxial minor veins					X		S		X						X									X		S	
Sometimes minor fiber bundle adjacent to the margin			X			X		X	X	X	X	X	X		X		X	X		X	X	X	X	X	X		X
Thick-walled fiber-like hypodermal cells protecting the margin		X																									

## Materials and methods

### Plants examined

Both live material and preserved herbarium material (Table 3) were used in this project. The living material used in this study came from the collections at Montgomery Botanical Center (MBC, Miami, FL). The dried material was from collections made while doing fieldwork in Brazil, from the garden and herbarium at Jardim Instituto Plantarum (HPL, Novo Odessa, São Paulo, Brazil) and from dried collections at the Fairchild Tropical Botanic Garden herbarium (FTG, Miami, FL) and a few specimens from the following herbaria: G, IBGE, IPA, K, MO, NY and US.

**Table 3.** Selected specimens examined.

***SYAGRUS* Martius**
*Syagrus allagopteroides* Noblick & Lorenzi, Lorenzi et al. 6792(HPL, FTG, NY, K, CEPEC, R, SP).
*Syagrus angustifolia* Noblick & Lorenzi, Lorenzi et al. 6636 (HPL, R, SP, BHCB, NY, K); Oliveira et al. 1082 (IBGE).
*Syagrus caerulescens* Noblick & Lorenzi, Lorenzi et al. 6649 (HPL, NY, K, R, SP); Tsuji & Franco 2622 (HPL).
*Syagrus campylospatha* (Barb. Rodr.) Becc., Hassler 1733 (G [holotype]), K, NY); Pedersen 14638 (G); Noblick et al. 5128 (FTG, PY, MBC96103).
*Syagrus cearensis* Noblick, Noblick et al. 4951 (EAC, FTG, RB, MBC94652); Noblick et al. 4953 (EAC, FTG, TEPB, MBC94654); Noblick et al. 5132 (IPA, MBC97262, MBC97263).
*Syagrus cerqueirana* Noblick & Lorenzi, Noblick et al. 5126(FTG, NY, PY, MBC96100); Schinini & Bordas 20288 (MO); Schinini & Bordas 20291 (CTES); Swallen 9520 (US).
*Syagrus cocoides* Mart., Fischer s.n. (MBC96363); Froes 11622 (GH, MO, NY); Henderson et al. 337 (NY); Henderson & Pardini 1503 (NY); Krukoff 1221 (F); Noblick 4954 (FTG, MBC94795); Plowman et al. 8267 (NY); Taylor et al.E1049 (NY).
*Syagrus coronata* (Mart.) Becc., Noblick & Soeiro 4694 (CEPEC, F, FTG, HRB, NY); Noblick & Soeiro 4704 (CEPEC, F, FTG, HRB); Noblick & Queiroz 4833 (FTG, HUEFS, MBC92146, MBC92196); Noblick et al. 4975 (FTG. IPA, MBC94473).
*Syagrus duartei* Glassman, Glassman & Gomes 8033 (F); Glassman 8035 (FTG); Hatschbach & Ferreira 35324 (MBM, F); Noblick 4854 (BHCB, FTG, MO, NY).
S. evansiana Noblick, Tsujiet et al. 2703 (HPL, R, BHCB, FTG, K, NY); Lorenzi 4269 (HPL); Lorenzi 4276 (FTG, HPL).
*Syagrus flexuosa* (Mart.) Becc., Noblick & Lima 4632 (BAH, CEPEC, CPATSA, F, FTG, NY, RB); Noblick & Lima 4633 (CEPEC, CPATSA, F, FTG); Noblick & Lima 4661 (CEPEC, CPATSA, F, FTG, NY); Noblick 4850 (BHCB, FTG, K); Noblick 4852 (BHCB, F, FTG, K, NY, US); Noblick & Ferreira 4869 (FTG, UFG); Noblick & Cropper 5108 (CEN, FTG, MBC96136); Noblick & Behr 5165 (IPA, MBC97800); Noblick & Behr 5166 (IPA, MBC97801); Noblick 5166 (IPA, MBC97801, MBC971463).
*Syagrus glaucescens* Glaziou ex Becc., Brown s.n. (MBC20030758); Glassman & Gomes 8112 (SP); Glassman 13002 (F, FTG); Noblick 4843 (BHCB, F, FTG, K, NY, US); Noblick 4845 (BHCB, FTG).
*Syagrus glazioviana* (Dammer) Becc., Noblick & Lobo 4527 (CEPEC, F, FTG, HRB, HUEFS, K, NY, RB, SP); 4617 (BAH, CEN, CEPEC, CPATSA, F, K, MICH, MO); Noblick & Lima 4643 (CEN, CEPEC, CPATSA, F, NY); Noblick & Lima 4659 (CPATSA, F, FTG), 4662 (CEPEC, CPATSA, F, FTG); Tsuji et al. 2681 (HPL)
*Syagrus gouveiana* Noblick & Lorenzi, *Lorenzi* 6537 (HPL, R, SP, BHCB, NY, K).
*Syagrus graminifolia* (Drude) Becc., Belem 2029 (UB); Burchell 5956 (K), holotype for *Cocos graminifolia* Drude; Davis & Shepherd 60024 (NY); Glassman 13093 (F); Noblick 5164 (FTG).
*Syagrus graminifolia* var. *glazioviana* (Dammer) Becc., Glaziou 22252 (G, K); Glaziou 22253 (G, K), isotype for *Cocos graminifolia* var. *glazioviana* Dammer; Lorenzi et al. 6791; Tsugi et al. 2682 (HPL).
*Syagrus harleyi* Glassman, Noblick 2867 (CEPEC, F, HUEFS, MO); Noblick & Lima 4380 (CEPEC, F, GH, HUEFS, MBM, SP); Noblick 4387 (BH, CEPEC, F, HUEFS, NY); Noblick 4389 (CEN, CEPEC, F, FTG, HRB, HUEFS, IPA, K, NY, RB, SP, US); Noblick & Lobo 4517 (AAU, ALCB, CEN, CEPEC, F, FTG, HRB, HUEFS, K, U).
*Syagrus itacambirana* Noblick & Lorenzi,Andrade-Lima 68-5425 (IPA); Tsuji et al. 2706 (HPL, R, SP, BHCB, NY, K).
*Syagrus kellyana* Noblick & Lorenzi, Noblick & Cline 5156(IPA, FTG, MBC97289, MBC97290).
*Syagrus lilliputiana* (Barb. Rodr.) Becc., Hassler 9519 (G); Lorenzi et al. 2805 (HPL).
*Syagrus loefgrenii* Glassman, Noblick & Lima 4634 (AAU, BAH, BH, CEPEC, CPATSA, F, FTG, K); Noblick & Lima 4660 (CPATSA, F, FTG); Noblick & Lima 4669 (ALCB, CEPEC, CPATSA, F, K, U); Lorenzi 6642 (HPL); Noblick & Buzeiro 4888 (BHCB, FTG, K, MO, US).
*Syagrus longipedunculata* Noblick & Lorenzi, Lorenzi et al. 6790 (HPL, R, SP, BHCB, NY, K); Oliveira et al. 588 (IBGE).
*Syagrus macrocarpa* Barb. Rodr., [No Collector] (MBC20080848, MBC20080849, MBC20080850); Noblick & Abrahao 4841 (BHCB, FTG, NY); Noblick & Abrahao 4842 (BHCB, FTG); Noblick 4857 (CESJ, F, FTG, IPA, NY, US).
*Syagrus mendanhensis* Glassman, Archer 4086 (BH [holotype], US); Glassman 13003 (FTG); Noblick 4844 (BHCB, F, FTG, MO, NY, K, US); Noblick 4846 (BHCB, FTG, NY, US); Noblick 4847 (BHCB, FTG).
*Syagrus microphylla* Burret, Glassman 13018031 (F, SP); Noblick & Clodoaldo 3508 (F, FTG, GH, HUEFS, MO, RB, SP); Noblick 4534 (ALCB, CEPEC, F, FTG, HUEFS, RB); Noblick & Lima 4612 (BAH, BH, CEPEC, CPATSA, F, FTG, K, NY, US); Noblick 4835 (FTG, MO).
*Syagrus minor* Noblick & Lorenzi, Lorenzi et al. 6639 (HPL, R, SP, BHCB, NY, K).
*Syagrus orinocensis* (Spruce) Burret, Balick et al. 1192 (NY); Betancur 1315 (NY); Bomm & Wentzel 6616 (NY); Davidse & Huber 15286 (BH); Mejia et al. 1258 (NY); Noblick et al. 4946 (FTG, MBC94586); Noblick et al. 4948 (FTG, PORT, MBC94588).
*Syagrus petraea* (Mart.) Becc., H. Lorenzi et al 6835 (HPL); Moreno 246 (JBSC); Saldias et al. 953 (NY).
*Syagrus pleioclada* Burret, Glassman & Gomes 8037042 (F, FTG [8037], SP [8041, 8042]); Hatschbach 35313 (F, MBM); Heringer & Castellanos SP80005 (SP); Martinelli & Smith 6333 (MO); Noblick 4853 (BHCB, FTG, MO, NY); Smith 6699 (US).
*Syagrus pleiocladoides* Noblick & Lorenzi, Lorenzi et. al. 6583 (HPL, R, SP, UB, UFMT, NY, FTG, K, AAU, CTES).
*Syagrus procumbens* Noblick & Lorenzi, Lorenzi et al. 6583 (HPL, R, SP, UB, UFMT, NY, FTG, K, AAU, CTES); Lorenzi 4752 (HPL); “emasensis” Noblick & Ferreira 4868 (FTG, UFG); Tsuji et al. 974 (HPL); Lorenzi et al. 6787 (HPL).
*Syagrus rupicola* Noblick & Lorenzi, Lorenzi et al. 6647 (HPL, R, SP, UB, NY, K).
*Syagrus stenopetala* Burret, *Liesner & Gonzalez 11928 (NY); Noblick & Smith 4936 (FTG, PORTO, MBC94576); Noblick & Smith 4938 (MBC94577); Pittier 9154 (NY, US); Steyermark* et al. *102432 (MO); Steyermark & Manara 110614 (BH).*
*Syagrus vagans* (Bondar) A. Hawkes, *Carvalho 2409* (CEPEC); Glassman & MedeirosCosta 8725726 (F); Lima & Noblick 140147 (CPATSA); Mori 10066 (CEPEC, NY); Noblick 3161 (HUEFS); Noblick et al. 3253 (HUEFS); Noblick & Clodoaldo 3537 (HUEFS); Noblick 3609 (HUEFS); Noblick 3846 (F, HUEFS).
*Syagrus vermicularis* Noblick, Fischer s.n. (MBC96364); Noblick & Feitosa 4971 (FTG, IPA); Noblick & Feitosa 4974 (FTG; MBC94690).
*Syagrus werdermannii* Burret, Carvalho 1790 (CEPEC, US); Glassman & MedeirosCosta 8728739 (F); Noblick & Clodoaldo 3769 (BH, F, HRB, HUEFS); Noblick & Lobo 4519 (BAH, CEN, CEPEC, F, FTG, HUEFS, K, MO, NY, RB).

### Anatomical preparation

Two methods were employed for expedient identification. First, one side of the middle section of a middle leaflet was folded back and forth on itself in accordion fashion; the folded leaflet was then held down on a cutting board, while using a double-edged razor blade to cut thin cross-sections. The sections were rinsed into a watch glass with water and a thin brush was used to select the thinnest sections under a dissecting scope and then placed on a microscope slide in a droplet of 1:1 glycerin/water solution. A cover glass was placed over the specimen and the slide was placed under a compound light microscope and photographed under the 10× objective (100× magnification). Most of the sections were unstained, but in rare cases toluline blue (0.01%) was tested to see if it made it easier to view certain characters, which it did not (Fig. 3C, 6D).

In the second method, better suited when material is limited, a small square of carrot of the appropriate size is cut to fit in an inexpensive hand-held student microtome. I purchase my hometrainingtools hand-held microtome online. A vertical slit is cut in the carrot and a small piece of leaflet is inserted in the appropriate orientation. The carrot is clamped into the hand-held microtome. The microtome is screwed to the appropriate level and an ordinary folding straight edge razor, the kind used for shaving, is utilized to cut the cross-sections and honed occasionally to keep it sharp. Sections are handled the same way as above. Scale was later added using a stage micrometer. Dried material can also be sectioned and photographed after rehydrating in a 5% solution of Contrad 70® (Decon Labs, King of Prussia, Pennsylvania) for a period of 24 hours (Tomlinson et al. 2011).

### Characters utilized

This paper is focused mainly on characters of the more easily sectioned marginal and laminal portions of the leaflet and not so much on the harder to section midrib. Trichomes, epidermis and dark staining bodies were also not looked at.

**Figure 2.**
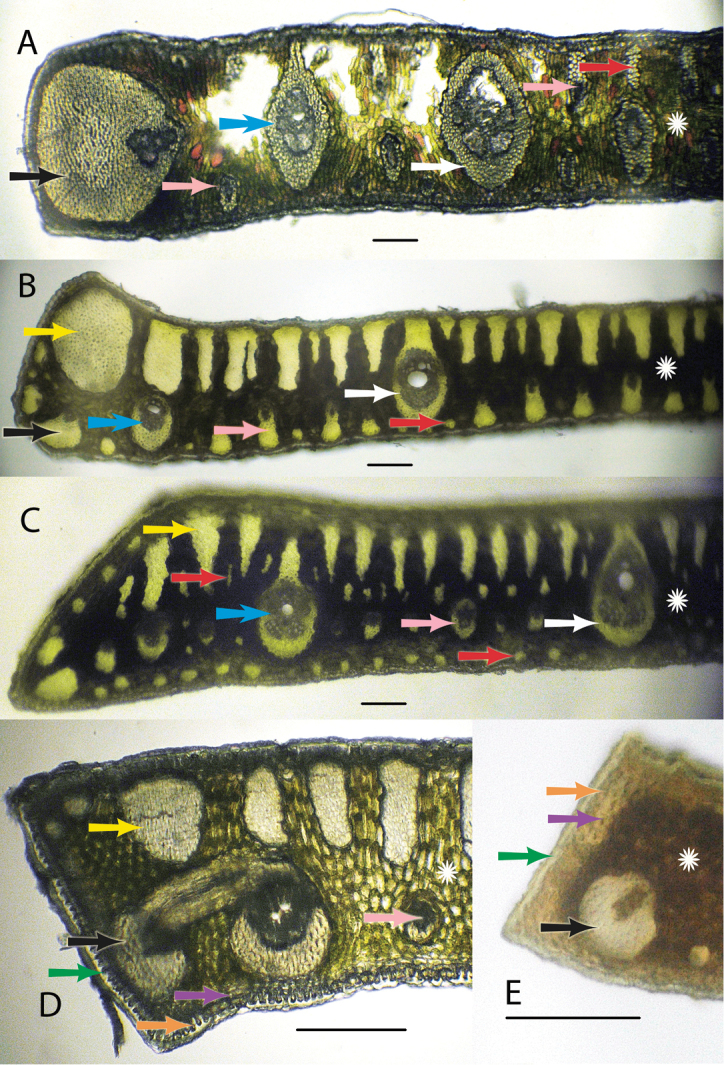
Anatomical characters. White arrows = major veins; Blue arrows = intermediate veins; Pink arrows = minor veins; Black arrows = vein with an exaggerated fibrous sheath; Yellow arrows = major fiber bundles; Red arrows = minor fiber bundles; Green arrows = Cuticle; Orange arrows = epidermis; Purple arrows = hypodermis; White star = mesophyll **A** *Syagrus allagopteroides* illustrates a large marginal vein with an exaggerated fibrous sheath (black), an unattached major vein (white), the presence of minor veins on both the adaxial and abaxial surfaces (pink) and an occasional minor adaxial fiber bundle (red) **B** *Syagrus caerulescens* illustrates a large marginal fiber bundle (yellow), a major vein attached to the adaxial surface by a fibrous extension (white), a small vein with an exaggerated fibrous sheath (black), minor veins (pink) sometimes alternating with minor fiber bundles (red) along the abaxial surface, and adaxial fiber bundles reaching nearly 1/2 the distance across the mesopyll (white star) **C** *Syagrus vagans* illustrates the first or second fiber bundle as being the largest along the adaxil surface (yellow) and minor fiber bundles (red) scattered throughout the mesophyll (white star), minor veins located near the middle or just slightly below **D** *Syagrus gouveiana* illustrates the cuticle (green), epidermis (orange), hypodermis (purple) **E** *Syagrus harleyi* illustrates a protective layer of thick-walled hypodermal cells (purple) on the margin, which is characteristic of this species. **A, B, C** Scale = 0.1 mm; **D, E** scale = 0.2 mm.

Characters examined during this study follow some of Glassman’s 4, 5 and 10 characters listed above and Tomlinson’s characters 4, 5, 6, and 7 listed above. Figure 2 will clarify much of the terminology and characters used in this paper. In each leaf cross-section the upper or superior side of the lamina is called the adaxial surface, meaning “towards the axis”, since this side of the leaf faces towards the axis or center of the plant as it grows out. The lower or inferior side is called the abaxial, meaning “away from the axis”, since this side faces away from the center of the plant (Dransfield et al. 2008, Esau 1977). The outer most layer of the leaf is the cuticle (Fig. 2D, 2E green arrow), a non cellular waxy layer produced by epidermis (Dransfield et al. 2008). The cuticle is followed by the epidermis, “outer skin” (Fig. 2D, 2E orange arrows), followed by the hypodermis, “under skin” (Fig. 2D, 2E purple arrows), which is finally followed by the mesophyll, “middle leaf”, region (Fig. 2 white stars). Within the mesophyll are vascular bundles, or fibrovascular bundles or veins of various sizes (Tomlinson et al. 2011) that will be referred to as major veins (Fig. 2 white arrows), intermediate veins (Fig. 2 blue arrows), and minor veins (Fig. 2 pink arrows). Some major and intermediate veins are often attached to the adaxial hypodermis and sometimes to both the surfaces by fibrous sheath extensions. If the attachment extends to both surfaces via a fibrous sheath extension, the vein becomes girder-like and is indeed referred to as a girder (Tomlinson et al. 2011) (Fig. 5A). In some veins the fibrous sheath becomes so enlarged with fibers that such veins are referred to as veins with exaggerated fiber sheaths (Tomlinson et al. 2011) (Fig. 2 black arrows). In addition to the veins, the laminal tissues are supported by nonvascular fibers or fiber bundles of various sizes. Some have major fiber bundles adjacent to or near their margins (Fig. 2 yellow arrows). Many fiber bundles are adaxial and may reach close to 1/2 the distance across the mesophyll (Fig. 2B, 2D). Minor, intermediate and major fiber bundles can be found adaxially (Fig. 2A red arrows; 2B, 2C yellow arrows). Most minor fiber bundles are mainly abundant abaxially (Fig. 2B, 2C red arrows) and occasionally scattered throughout the mesophyll (Fig. 2C red arrows).

**Figure 3.**
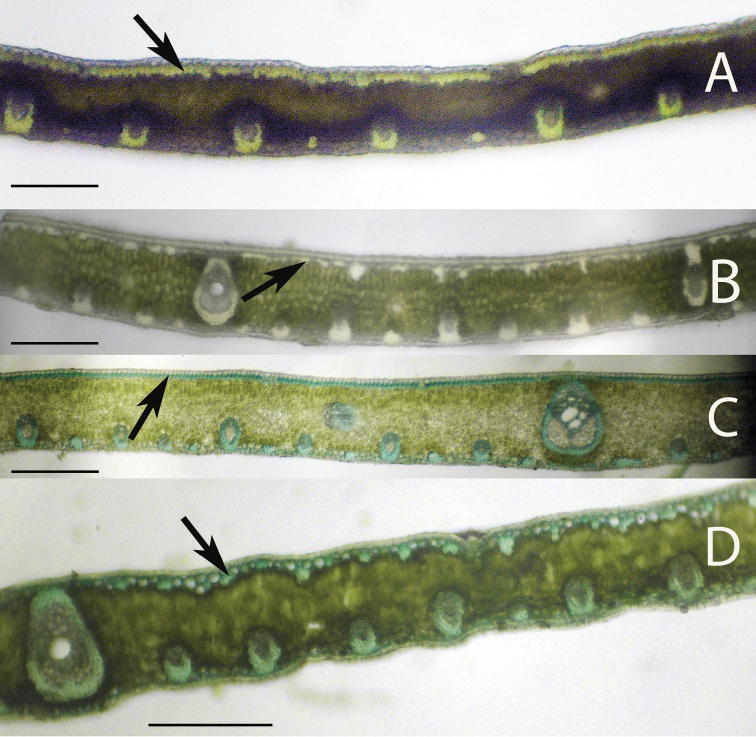
Leaflet cross-sections of the Rain Forest Clade of *Syagrus* species with arrows pointing out the 1–2 cell thick fibrous sheet just below the epidermis that is a defining character of species of this clade: **A** *Syagrus vermicularis* Noblick **B** *Syagrus stenopetala* Burret **C** *Syagrus sancona* (Mart.) Becc **D** *Syagrus cocoides* (Mart.) Mart. Scale = 0.2 mm.

To keep things simple for field examination, the following qualitative characters were examined: (1) location, attachment or lack of attachment of the major veins to one or both surfaces and method of attachment (fiber sheath extension or not); (2) location, attachment or unattachment of intermediate veins to one or both surfaces and method of attachment (e.g. sheath extensions, formation of girders); (3) location of the minor veins (e.g. adaxial, abaxial, abaxial and adaxial, middle, marginal); (4) presence, size and location of veins with an exaggerated fibrous sheath (large ones often located on the leaflet margin); (5) presence, location, size and sometimes cross-sectional shape of fiber bundles and the extent they reach across the mesophyll. These characters can also be further summarized as follows:

(1) Major vein location {adjacent to the margin; near the margin but not adjacent to it (this means that along a horizontal plane there is a maximum of one minor vein or one fiber bundle separating it from the actual margin); not adjacent to nor near the margin}
(2) Major vein attached where {unattached; attached to adaxial hypodermal surface only; attached to both adaxial and abaxial hypodermal surfaces}
(3) Major vein attachment how{attached by a short or long fibrous sheath extension; attachment not by a fibrous sheath extension}
(4) Intermediate veins attached {unattached; attached to adaxial surface only; attached to both surfaces}
(5) Intermediate vein attachment {to both surfaces by fibrous sheath extension (girders); attached to adaxial surface only by fibrous sheath extension; attached but without fibrous sheath extension}
(6) Minor vein location {adjacent to both the adaxial and abaxial surface; a few adjacent to the adaxial but most on the abaxial surface; near the middle of the mesophyll; adjacent to the abaxial surface or at least in the lower third of the mesophyll; only adjacent to the abaxial surface}
(7) Presence of major marginal vein with large exaggerated fibrous sheath {absent; present}
(8) Presence of minor marginal vein with exaggerated fibrous sheath {absent; one present; two or more present}
(9) One major rounded fiber bundle adjacent to the margin {absent; present}
(10) First fiber bundle on the adaxial surface the largest {absent; present}
(11) Adaxial fiber bundles size if present {reach 1/3 to 1/2 across the mesophyll; reach 1/5 to 1/4 across the mesophyll}
(12) Fiber bundles shape {mostly long and skinny: mostly long and thick: mostly short and thick}
(13) Fibers or minor fiber bundle locations {adaxial only; adaxial and abaxial only; adaxial, abaxial and scattered in the mesophyll}
(14) Minor fiber bundles adjacent to the margin {absent; present}.
(15) Minor fiber bundles abundance {none; few along the adaxial and abaxial surface; only a few along the abaxial surface alternating with the minor veins; many along the abaxial surface}
(16) Thick walled hypodermis protecting the margin {absent; present}

The key was designed for field use, which means minimal equipment, no staining, and low magnification and the use of simple characters. Refer to the characters in the methods for clarification of terminology. By using the methods listed above and following many of the simple techniques mentioned by Tomlinson et al. (2011), rapid results can be achieved in a laboratory provided with only the simplest equipment. This simple approach was also successfully used in a significant study of palm leaf development by Nowak et al. (2007).

## Results

**Figure 4.**
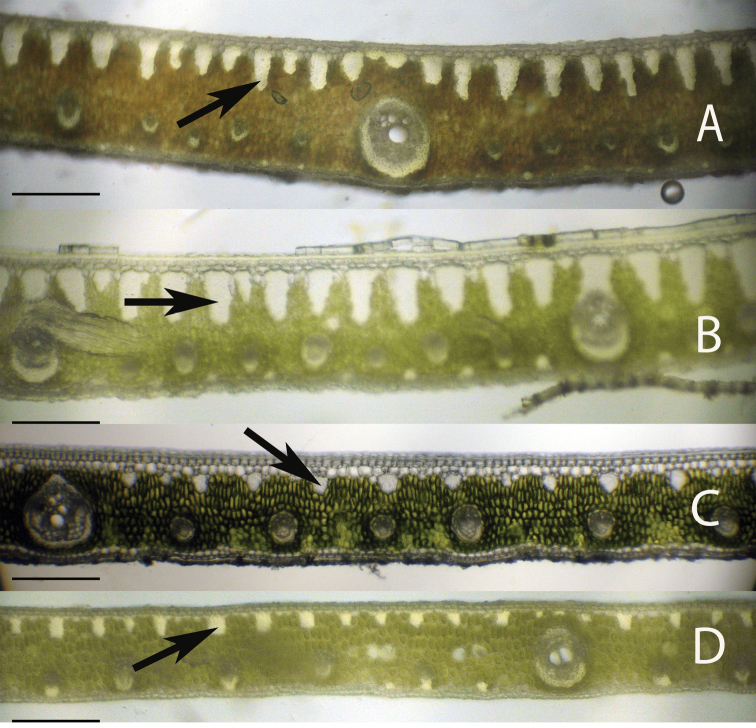
Leaflet cross-sections of the Eastern Brazilian Clade of *Syagrus* species with arrows showing the multicellular fiber bundles that are a defining character of species of this clade: **A** *Syagrus coronata* (Mart.) Becc. **B** *Syagrus glaucescens* Glaziou & Becc. **C** *Syagrus kellyana* Noblick & Lorenzi **D** *Syagrus cearensis* Noblick. Scale = 0.2 mm.

**Figure 5.**
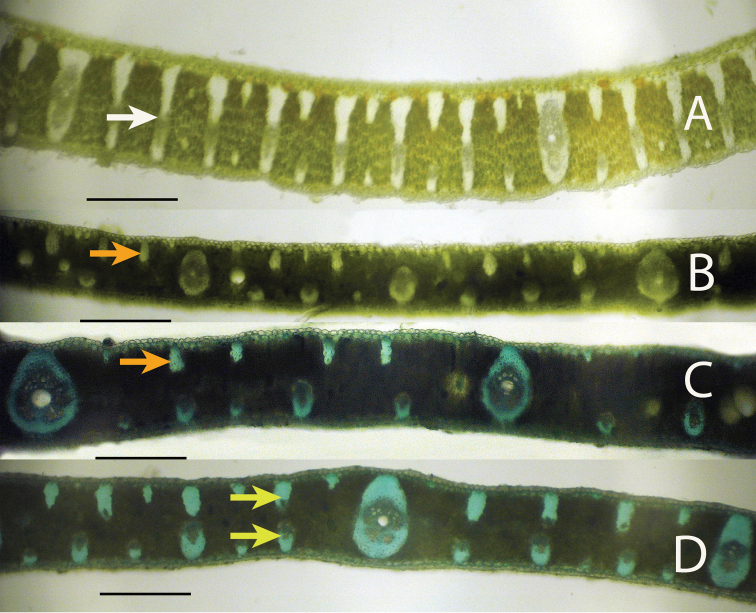
Leaflet cross-sections of the Cluster-stem Clade of *Syagrus* species. (A) *Syagrus campylospatha*, white arrow pointing at an intermediate vein with fibrous sheath extensions to both surfaces forming a girder type vein **B** *Syagrus macrocarpa* Barb. Rodr., orange arrow indicating a minor fiber bundle **C** *Syagrus flexuosa* (Mart.) Becc. orange arrow indicating a minor fiber bundle **D** *Syagrus cerqueirana*, yellow arrows indicating minor veins on both surfaces of the leaflet. Scale = 0.2 mm.

Anatomical characters observed on marginal palm leaflet cross-sections have been found to verify the Rain Forest and Eastern Brazilian clades and to some extent the Cluster-stem clade found within *Syagrus* (Fig. 1). In the Rain Forest clade, there is a continuous hypodermal layer of fibrous to thick-walled cells, one to two layers thick just below the adaxial epidermis (Fig. 3A, 3B, 3C, 3D). In the Eastern Brazilian clade, there are many thick, closely-spaced, multicellular fiber bundles running along the adaxial surface of the leaflet (Fig. 4A, 4B, 4C, 4D). Finally the Cluster-stem clade is usually characterized by minor sparsely spaced fiber bundles on the adaxial side and minor veins adjacent to the abaxial surface (Fig. 5B, 5C) or with minor veins on both surfaces (Fig. 5D) that make the anatomy of *Syagrus macrocarpa* Barb. Rodr. *Syagrus flexuosa* (Mart.) Becc. and *Syagrus cerqueirana* look interestingly similar to one another.

**Figure 6.**
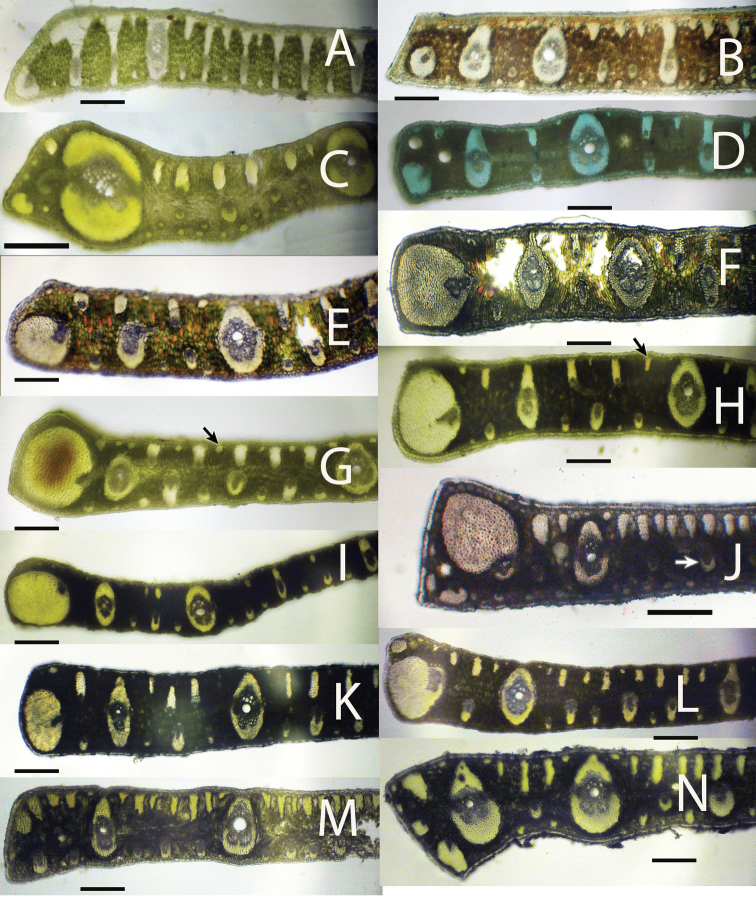
Leaflet cross-sections of acaulescent *Syagrus* species found in the key: **A** *Syagrus campylospatha* **B** *Syagrus harleyi* **C** *Syagrus procumbens* “emasensis” **D** *Syagrus cerqueirana* **E** *Syagrus minor* **F** *Syagrus allagopteroides* **G** *Syagrus lilliputiana*, arrow indicates a rounded minor fiber bundle **H** *Syagrus cerqueirana*, arrow indicate an elongated, longer than wide minor fiber bundle **I** *Syagrus loefgrenii* **J** *Syagrus longipedunculata*, arrow indicates a minor vein located in the middle of the mesophyll **K** *Syagrus angustifolia* **L** *Syagrus itacambirana* **M** *Syagrus petraea* **N** *Syagrus procumbens*, note major vein near but not adjacent to the margin. Scale = 0.2 mm.

After examining many leaflet hand sections of various acaulescent palm specimens, it was discovered that many had very different leaflet anatomy. The presence and absence of the anatomical characters in all 25 species is recorded in Table 2. Useful anatomical characters were found to separate the 25 known species of acaulescent and short-stemmed *Syagrus* and an identification key was developed. Several acaulescent *Syagrus* specimens frequently identified as *Syagrus petraea* were found to have distinctive field habits and leaflet anatomies (Table 1 and 2).

## Discussion

### Distinguishing Major Clades

Species of the Rain Forest clade (Fig. 1), which includes many Amazonian species, are distinguished anatomically by an almost continuous adaxial fibrous layer, one or a few cells thick just under the epidermis (the hypodermal layer) (Fig. 3A, 3B, 3C, 3D). I speculate that perhaps this nearly continuous fibrous layer strengthens the lamina while maintaining its flexibility (Vincent 1982), helps the leaf shed water and discourages fungus infection. Species of the Eastern Brazilian clade (Fig. 1) are distinguished by thicker and stiffer leaflets reinforced by many adaxial, thick, multicellular fiber bundles along the adaxial side of the leaf and these fiber bundles may extend as far as 1/2 the distance across the mesophyll (Fig. 4A, 4B, 4C, 4D). Fibers assume much of the load-bearing capacity of the lamina (Horn et al. 2009, Vincent 1982). These fibers and fiber bundles help the leaflet to retain its shape, flexibility and form when leaf turgidity wanes during the periodic dry spells that frequent the seasonally dry forests, *cerrados* and *caatingas* of Eastern Brazil. The Cluster-stem clade (perhaps a misnomer, since not all are cluster-stemmed) is not as clear. *Syagrus campylospatha* (Figs 5A, 6A) appears anatomically different from the rest of the group having girder type intermediate veins that are attached to both sides of the leaf by fibrous sheath extensions. *Syagrus flexuosa* and *Syagrus macrocarpa* have nearly identical anatomies with minor, sparsely spaced fiber bundles running along the adaxial surface (Fig. 5B, 5C) and *Syagrus cerqueirana* (formerly identified as *Syagrus petraea*) has a similar aspect but replaces these adaxial fiber bundles with minor veins, which are found on both sides of the leaf (Fig. 5D).

**Figure 7.**
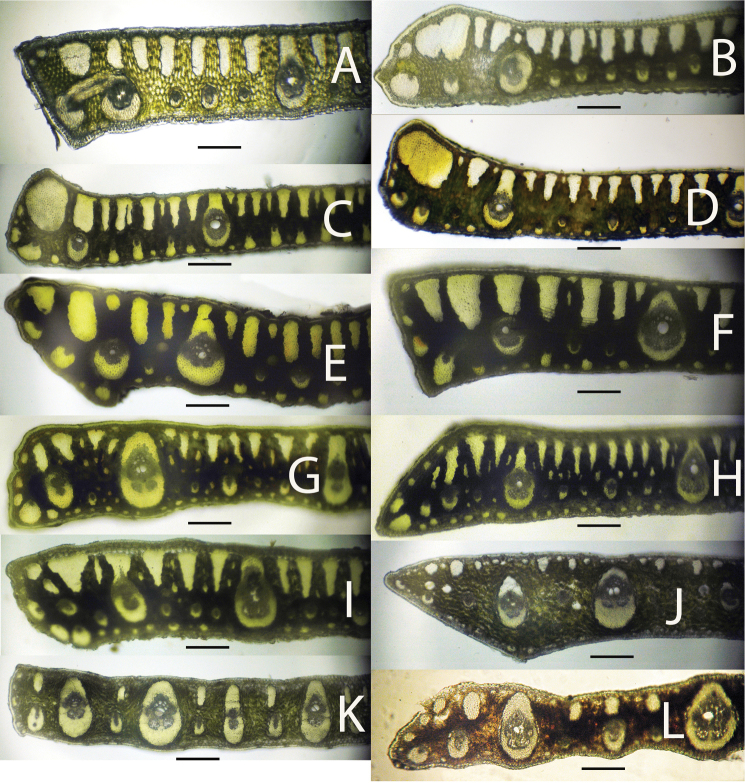
Leaflet cross-sections of acaulescent *Syagrus* species found in the key: **A** *Syagrus gouveiana* **B** *Syagrus drudei* **C** *Syagrus caerulescens* **D** *Syagrus rupicola* **E** *Syagrus glazioviana* **F** *Syagrus evansiana* **G** *Syagrus werdermanii* **H** *Syagrus vagans* **I** *Syagrus microphylla* **J** *Syagrus pleiocladoides* **K** *Syagrus pleioclada* **L** *Syagrus graminifolia* **M** *Syagrus mendanhensis* **N** *Syagrus graminifolia* var. *glazioviana*. Scale = 0.2 mm.

Most acaulescent *Syagrus* exhibit the Eastern Brazilian pattern (e.g. *Syagrus gouveiana*; Fig. 7A) with the large, multicellular fiber bundles running along the adaxial side of the leaflet and the Cluster-stem pattern, similar to that of *Syagrus cerqueirana* (Fig. 5D, 6D, 6H), with minor veins on both surfaces (e.g. *Syagrus lilliputiana*,Fig. 6G), each attached to either the adaxial or abaxial surface by short, fibrous extensions. Since most acaulescent palms grow in seasonally dry areas (*cerrados*) that require stiffer leaflets, it is perhaps understandable why the Rain Forest pattern is not seen among them.

### Acaulescent species

Some of the problems of identifying acaulescent *Syagrus* species were covered previously in the introduction concerning the lack of good label information in relation to how leaves and leaflets are displayed or arranged on the plant before pressing and drying. Having observed most of these variations personally in the field has led me to the challenging process of trying to straighten out this much neglected complex of species. For me, it started in Bahia, Brazil with the misidentification of the acaulescent *cerrado* palm, *Syagrus glazioviana*. Many palm taxonomists, including Glassman and myself (Noblick 1991), have erroneously identified it as *Syagrus petraea*. Initially, Glassman (1965) placed it in synonomy with *Syagrus petraea*, based on the shape and size of their female flowers. A few years later, he considered them distinct species (Glassman 1968) after he had seen the lectotype for *Syagrus glazioviana* due to differences in the width of the leaflets and shape of the leaflet tips (symmetrical verses asymmetrical). Finally in his revision (Glassman 1987), he synonomized it once again with *Syagrus petraea*, concluding that the differences seen must have been due to favorable versus unfavorable growing conditions. In western Bahia it is often used to make brooms. It has meter-long leaves with long, regular to loosely clustered pinnae and a spike inflorescence. In the same *cerrados*, one will encounter another acaulescent, spicate palm with shorter leaves and tightly clustered pinnae that are unsuitable for broom making, which I had previously identified as *Syagrus petraea* as well. I currently believe that the smaller western Bahian one is *Syagrus loefgrenii*, which has also been proposed as a synonym of *Syagrus petraea* (Henderson et al. 1995), but the leaflet anatomy of *Syagrus petraea* (Fig. 6M) is very different from the anatomy of both *Syagrus loefgrenii* (Fig. 6I) and *Syagrus glazioviana* (Fig. 7E). It has been an unfortunate fact that most acaulescent, spicate *Syagrus* have gotten automatically classified as *Syagrus petraea*. In truth, acaulescent palms with a sparsely branched inflorescence have also been classified as *Syagrus petraea* (i.e. *Syagrus cerqueirana* from Paraguay). However *Syagrus petraea* is not the only problematic acaulescent *Syagrus*. In his anatomy paper (Glassman 1972), Glassman misidentified *Syagrus cerqueirana* (Swallen 9520 from Mato Grosso) as *Syagrus graminifolia*. He then proceeded to use the anatomy of that misidentified specimen to represent *Syagrus graminifolia* in his publications (Glassman 1972, 1987). When I examined the anatomy of collections of *Syagrus graminifolia* from Goiás I discovered that they had a very different anatomy from what Glassman had published, but I resolved the issue by comparing the anatomy of my specimens with that of a leaflet fragment borrowed from the original 1827 holotype (Burchell 5956) and found them to be a match. In addition, Glassman mistakenly reported Burchell’s collection from the state of Piauí, but Burchell’s field notes and itinerary (Smith and Smith 1967) clearly place him in southern Goiás at the time.

Many of the *Syagrus petraea*-types have very different leaflet anatomies. Their visible field characters (Table 1) and their distinctive anatomy has justified splitting up the complex (Noblick and Lorenzi 2010; Lorenzi et al. 2010) by resurrecting formerly synonymized names (e.g. *Syagrus glazioviana*, *Syagrus loefgrenii*) and by describing several new species (e.g. *Syagrus allagopteroides*, *Syagrus angustifolia*, *Syagrus caerulescens*, *Syagrus cerqueirana*, *Syagrus evansiana*, *Syagrus gouveiana*, *Syagrus itacambirana*, *Syagrus minor*, *Syagrus pleiocladoides*, *Syagrus procumbens*, *Syagrus rupicola*). Currently, there are about 26 taxa of *Syagrus* without visible above ground stems or with very short stems and it is strongly suspected that there will be several more based on the anatomy that has so far been observed. As a disclaimer, I feel that this key is still not the final word and will need further revision as new species are discovered and others get reworked. The same species grown under different growing conditions or adult and juvenile forms may look slightly different, but the anatomy maybe an important tool in helping us to resolve these issues. I suspect that some species which appear to be morphologically different but anatomically similar may turn out to be the same species (e.g. *Syagrus allagopteroides* and *Syagrus minor* are suspicious).

In conclusion, leaflet anatomy has been found to be useful in helping to confirm or verify relationships discovered through the molecular analysis and in identifying some of the difficult acaulescent *Syagrus* species.

### Anatomical key to acaulescent *Syagrus*

**Table:** 

1	Many large intermediate veins with fibrous extensions to both adaxial (upper) and abaxial (lower) surfaces forming girders across the leaflet (Fig. 5A, 6A)	*Syagrus campylospatha*
–	No such girders formed	2
2	Margin of leaflet protected by a layer of thick-walled cells (Fig. 2E, 6B)	*Syagrus harleyi*
–	Margin of leaf lacking protective layer with few fibers, veins with exaggerated fibrous sheaths, large fibrous bundles	3
3	Margin with a huge fully functional major vein with a somewhat exaggerated fibrous sheath at or near the margin (Fig. 6C)	*Syagrus procumbens*“emasensis”
–	Margin with a vein with an exaggerated fibrous sheath, fiber bundles or anything other than a major vein	4
4	Minor veins adjacent to both the adaxial and abaxial surface (Fig. 6D-H)	5
–	Minor veins mostly present adjacent to the abaxial surface and few if any on the adaxial surface	9
5	A minor to intermediate vein with an exaggerated fibrous sheath adjacent to the margin and occupying less than half of the margin (Fig. 6D)	*Syagrus cerqueirana*
–	A major vein with an exaggerated fibrous sheath adjacent to the margin and occupying more than half to nearly the entire margin (Fig. 2A)	6
6	Marginal vein with exaggerated fibrous sheath occupies over half of the margin but not the entire margin (Fig. 6E)	*Syagrus minor*
–	Marginal vein with exaggerated fibrous sheath occupies the entire margin	7
7	Major vein usually unattached separated from the hypodermis by another cell layer or two (Fig. 2A, 6F)	*Syagrus allagopteroides*
–	Major vein usually attached to the adaxial hypodermis but separated from the abaxial by an additional cell layer or two	8
8	Minor fiber bundles along the adaxial nearly round in shape (Fig. 6G)	*Syagrus lilliputiana*
–	Minor fiber bundles along the adaxial elongated, longer than wide (Fig. 6H)	*Syagrus cerqueirana*
9	Vein with a very large exaggerated fibrous sheath adjacent to the margin	10
–	Margin without such a vein but with or without minor veins, and/or minor or major large fiber bundles	13
10	Minor veins near the middle of the mesophyll (Fig. 6J)	*Syagrus longipedunculata*
–	Minor veins adjacent to the lower abaxial surface	11
11	Most large adaxial fiber bundles reaching less than 1/4 to 1/5 across the mesophyll (Fig. 6I)	*Syagrus loefgrenii*
–	Most large adaxial fiber bundles reach 1/3 to 1/2 across the mesophyll	12
12	A few minor veins near or attached to the adaxial surface and veins often alternating with the minor fiber bundles adjacent to the abaxial surface (Fig. 6K)	*Syagrus angustifolia*
–	No minor veins near or attached to the adaxial surface and minor veins but no fiber bundles present on the abaxial surface (Fig. 6L)	*Syagrus itacambirana*
13	Large major vein near the margin but not adjacent to it (Fig. 6N)	*Syagrus procumbens*
–	Major veins neither near the margin nor adjacent to it	14
14	Margin with one very large fiber bundle or the first or second adaxial fiber bundles are larger than the rest	15
–	Margin with no significantly large fiber bundles	24
15	Adaxial fiber bundles long and skinny and reaching less than 1/5 to ¼ across the mesophyll (Fig. 6M)	*Syagrus petraea*
–	Adaxial fiber bundles long and usually fat and reaching 1/3 to 1/2 across the mesophyll	16
16	No minor fiber bundles scattered throughout the mesophyll	17
–	Minor fiber bundles scattered throughout the mesophyll	22
17	Minor fiber bundles usually absent from the abaxial surface	18
–	Minor fiber bundles usually present either along the abaxial surface and/or margin	19
18	Major veins are usually attached adaxially (Fig. 2D, 7A)	*Syagrus gouveiana*
–	Major veins are usually unattached (Fig. 7B)	*Syagrus duartei*
19	One major fiber bundle adjacent to the margin	20
–	No major fiber bundle adjacent to the margin	21
20	Minor veins all attached to the adaxial hypodermis (Fig. 2B, 7C)	*Syagrus caerulescens*
–	Some adaxial minor veins attached but many unattached (Fig. 7D)	*Syagrus rupicola*
21	Major vein attached to adaxial surface by a fibrous sheath extension (Fig. 7E)	*Syagrus glazioviana*
–	Major vein usually unattached (Fig. 7F)	*Syagrus evansiana*
22	Major veins surrounded by a very thick fibrous sheath (Fig. 7G)	*Syagrus werdermanii*
–	Major veins surrounded by a thin to medium fibrous sheath	22
23	Adaxial fiber bundles mostly long and skinny in cross-section (Fig. 2C, 7H)	*Syagrus vagans*
–	Adaxial fiber bundles mostly long and thicker (Fig. 7I)	*Syagrus microphylla*
24	Most adaxial fiber bundles reach less than 1/5 across the mesophyll (Fig. 7J)	*Syagrus pleiocladoides*
–	Most adaxial fiber bundles reach 1/3 to 1/2 across the mesophyll	25
25	Leaflets deflexed, adaxial fiber bundles more rounded (Fig. 7L)	*Syagrus pleioclada*
–	Leaflets straight or erect, adaxial fiber bundles long and skinny	26
26	Leaflets silvery blue color, very small minor fiber bundles between the veins and intermediate fiber bundles adaxially, and a few minor fibers abaxially (Fig. 7L)	*Syagrus graminifolia*
–	Leaflets green in color, no or few small minor fiber bundles between the veins and intermediate fiber bundles adaxially and none abaxially	27
27	Leaflets with many minor fibers adjacent to the margin (Fig. 7M)	*Syagrus mendanhensis*
–	Leaflets with one to no minor fiber bundles adjacent to the margin (Fig. 7N)	*Syagrus graminifolia* var. *glazioviana*

## Appendix

Numerical list to *Syagrus* taxa and numbered collections. (doi: 10.3897/phytokeys.26.5436.app) File format: Microsoft Word file (doc).
