# Supplementary material for: Leaflet anatomy verifies relationships within Syagrus (Arecaceae) and aids in identification
Source: PhytoKeys. 2013 Sep 30;(26):75–99. doi: 10.3897/phytokeys.26.5436 (PMC3817423; doi:10.3897/phytokeys.26.5436)
Supplement: Supplementary file 1 — Numerical list to Syagrus taxa and numbered collections. (doi: 10.3897/phytokeys.26.5436.app) File format: Microsoft Word file (doc). [file PhytoKeys-026-075-s001.doc]

**NUMERICAL LIST TO SYAGRUS TAXA (doi: 10.3897/phytokeys.@.5436)**

1. *S. allagopteroides* Noblick & Lorenzi
2. *S. angustifolia* Noblick & Lorenzi
3. *S. caerulescens* Noblick & Lorenzi
4. *S.* *campylospatha* (Barb. Rodr.) Becc.
5. *S. cearensis* Noblick
6. *S. cerqueirana* Noblick & Lorenzi
7. *S*. *cocoides* Mart.
8. *S*. *coronata* (Mart.) Becc.
9. *S*. *duartei* Glassman
10. *S. evansiana* Noblick
11. *S. flexuosa* (Mart.) Becc.
12. *S*. *glaucescens* Glaziou ex Becc.
13. *S. glazioviana* (Dammer) Becc.
14. *S. gouveiana* Noblick & Lorenzi
15. *S*. *graminifolia* (Drude) Becc.
16. *S*. *graminifolia* var. *glazioviana* (Dammer) Becc.
17. *S*. *harleyi* Glassman
18. *S. itacambirana* Noblick & Lorenzi
19. *S. kellyana* Noblick & Lorenzi
20. *S*. *lilliputiana* (Barb. Rodr.) Becc.
21. *S*. *loefgrenii* Glassman
22. *S. longipedunculata* Noblick & Lorenzi
23. *S*. *macrocarpa* Barb. Rodr.
24. *S*. *mendanhensis* Glassman
25. *S*. *microphylla* Burret
26. *S. minor* Noblick & Lorenzi
27. *S*. *orinocensis* (Spruce) Burret
28. *S*. *petraea* (Mart.) Becc.
29. *S. pleioclada* Burret
30. *S. pleiocladoides* Noblick & Lorenzi
31. *S. procumbens* Noblick & Lorenzi
32. *S. rupicola* Noblick & Lorenzi
33. *S*. *stenopetala* Burret
34. *S. vagans* (Bondar) A. Hawkes
35. *S. vermicularis* Noblick
36. *S*. *werdermannii* Burret

**INDEX TO NUMBERED COLLECTIONS**

Abrahao 3 (17)

Anderson 7175 (11)

Anderson 7939 (11)

Anderson 8178 (11)

Andrade s.n. (BHCB 8883, 8885)(9)

Andrade‑Lima 73‑7371 (7)

Archer 4078 (12)

Archer 4086 (24)

Balee 1920 (7)

Balick 1192 (27)

Balick 1345 (7)

Barreto 1206 (29)

Barreto 4824 (11)

Belem 2029 (15)

Belem 466–467 (11)

Belem 493 (11)

Betancur 1315 (27)

Blydenstein 1688 (27)

Bockermann 240 (7)

Bomm 6616 (27)

Brown s.n. (MBC20030758)(12)

Calderon 2418 (17)

Campbell P22409 (7)

Campos Porto RB79194 (23)

Carvalho 1790 (36)

Carvalho 2000 (8)

Carvalho 2409 (34)

Cid 522 (7)

Custodio Filho 493 (11)

Cytryn 3 (11)

Cytryn 9 (12)

Dambros 239 (11)

Davidse 15286 (27)

Davis 60024 (15)

Duarte 2018 (9)

Duarte 2019 (29)

Duarte 9341 (11)

Duarte 9341 (RB)

Duarte 9603 (25)

Eiten 5449 (7)

Eiton 1478 (11)

Fernandes 2008 (23)

Fernandes 2885 (23)

Fernandes 2894 (23)

Ferri SP49327 (11)

Filho 146 (11)

Fischer s.n. (MBC96363)(7)

Fischer s.n. (MBC96364)(35)

Forest Dep. Br. Guiana G560‑7575 (7)

Freitas s.n. (BHCB 1280, 13298)(9)

Froes 11622 (7)

Gentry 21431 (11)

Gentry 49964 (8)

Glassman 13002 (12)

Glassman 13003 (24)

Glassman 13018–13031 (25)

Glassman 13037–13040 (11)

Glassman 13043–13046 (13)

Glassman 13085 (SP)

Glassman 13093 (15)

Glassman 8002 (11)

Glassman 8011–8015 (21)

Glassman 8033 (9)

Glassman 8035 (9)

Glassman 8037–8042 (29)

Glassman 8112 (12)

Glassman 8723 (8)

Glassman 8725–8726 (34)

Glassman 8728–8739 (36)

Glassman 8750 (11)

Glaziou 15559 (11)

Glaziou 19999 (11)

Glaziou 20024 (12)

Glaziou 20026 (11)

Glaziou 22252 (15)

Glaziou 22253 (16)

Glaziou 22261 (11)

Glaziou 8058 (8)

Glaziou 9015 (23)

Goodland 380 (383?) (21)

Goodland 725 (21)

Goodland 900 (21)

Gottsberger 1060 (21)

Harley 18657 (11)

Harley 18696 (17)

Harley 19340 (25)

Harley 19411 (8)

Harley 21351 (11)

Harley 22816 (25)

Hassler1733 (4)

Hassler 9519 (20)

Hatschbach 35313 (29)

Hatschbach 35324 (9)

Hatschbach 36872 (11)

Hatschbach 37477 (11)

Hatschbach 44102 (13)

Hatschbach 48000 (17)

Henderson 1503 (7)

Henderson 337 (7)

Heringer 10408 (11)

Heringer 10475 (11)

Heringer 1780 (11)

Heringer 18329 (11)

Heringer 2580 (11)

Heringer SP80005 (29)

Horst 61 (12)

Horst 65 (29)

Horst 68 (11)

Horst 74 (9)

Irwin 11578 (11)

Irwin 12766 (11)

Irwin 15458 (11)

Irwin 26637 (11)

Irwin 27003 (11)

Irwin 27605 (12)

Irwin 32833 (11)

Irwin 5296 (11)

Irwin 6313 (11)

Kirizawa 263 (11)

Kirizawa s.n. (SP 204268) (11)

Krukoff 1221 (7)

Kuhlmann 1572 (21)

Kuhlmann 3964 (21)

Kuhlmann 4541 (21 [one 11 infl.])

Kuhlmann 6313 (12)

Liesner 11928 (33)

Lima 1 (8)

Lima 108–117 (17)

Lima 128–139 (25)

Lima 140–147 (34)

Lima 99–107 (11)

Lorenzi 4269 (10)

Lorenzi 4276 (10)

Lorenzi 4752 (31)

Lorenzi 6537 (14)

Lorenzi 6583 (30)

Lorenzi 6583 (31)

Lorenzi 6636 (2)

Lorenzi 6639 (26)

Lorenzi 6642 (21)

Lorenzi 6647 (32)

Lorenzi 6649 (3)

Lorenzi 6787 (31)

Lorenzi 6790 (22)

Lorenzi 6792 (1)

Lorenzi 6835 (28)

Lyra 463 (8)

Lyra 659 (8)

Lyra Lemos 1048 (8)

Lyra Lemos 1102 (8)

Lyra Lemos 1812 (8)

Lyra Lemos 1822 (34)

Macedo 3324 (11)

Macedo 496 (11)

Mantovani s.n. (SP 162522)( 29)

Martinelli 5319 (17)

Martinelli 5918 (12)

Martinelli 6333 (29)

Martius (F‑neg. 18563 & 18563a) (8)

Mattos 12291 (11)

McKenzie C22 (7)

Medeiros‑Costa 145 (11)

Medeiros‑Costa 153–0161 (21)

Medeiros‑Costa 183–0189 (21)

Medeiros‑Costa 2 (8)

Medeiros‑Costa 261 (13)

Medeiros‑Costa 261 (21)

Mejia 1258 (27)

Moreno 246 (28)

Mori 10000 (8)

Mori 10066 (34)

Mori 12305 (8)

Mori 12638 (17)

Mori 13124 (11)

Mori 13328 (11)

Noblick 2071 (8)

Noblick 2821 (11)

Noblick 2867 (17)

Noblick 3161 (34)

Noblick 3253 (34)

Noblick 3508 (25)

Noblick 3537 (34)

Noblick 3609 (34)

Noblick 3769 (36)

Noblick 3787 (11)

Noblick 3846 (34)

Noblick 4379 (17)

Noblick 4380 (17)

Noblick 4386 (11)

Noblick 4387 (17)

Noblick 4388 (17)

Noblick 4389 (17)

Noblick 4513 (8)

Noblick 4516 (11)

Noblick 4517 (17)

Noblick 4519 (36)

Noblick 4521 (13)

Noblick 4527 (13)

Noblick 4534 (25)

Noblick 4592 (11)

Noblick 4611 (25)

Noblick 4612 (25)

Noblick 4616– 4617 (13)

Noblick 4619 (11)

Noblick 4624 (13)

Noblick 4626 (11)

Noblick 4630 (11)

Noblick 4632 (11)

Noblick 4633 (11)

Noblick 4634 (21)

Noblick 4636 (13)

Noblick 4637 (13)

Noblick 4643 (13)

Noblick 4652 (21)

Noblick 4653 (11)

Noblick 4655 (21)

Noblick 4659 (13), 4662 (13)

Noblick 4660 (21)

Noblick 4661 (11)

Noblick 4665 (21)

Noblick 4669 (21)

Noblick 4688 (8)

Noblick 4694 (8)

Noblick 4704 (8)

Noblick 4833 (8)

Noblick 4835 (25)

Noblick 4841 (23)

Noblick 4842 (23)

Noblick 4843 (12)

Noblick 4844 (24)

Noblick 4845 (12)

Noblick 4846 (24)

Noblick 4847 (24)

Noblick 4850 (11)

Noblick 4852 (11)

Noblick 4853 (29)

Noblick 4854 (9)

Noblick 4857 (23)

Noblick 4858 (11)

Noblick 4868 (31 “emasensis”)

Noblick 4869 (11)

Noblick 4888 (21)

Noblick 4892 (8)

Noblick 4936 (33)

Noblick 4938 (33)

Noblick 4946 (27)

Noblick 4948 (27)

Noblick 4951 (5)

Noblick 4953 (5)

Noblick 4954 (7)

Noblick 4971 (35)

Noblick 4974 (35)

Noblick 4975 (8)

Noblick 5108 (11)

Noblick 5126 (6)

Noblick 5128 (4)

Noblick 5132 (5)

Noblick 5156 (19)

Noblick 5165 (11)

Noblick 5166 (11)

Normanha s.n. (SP 44403)(11)

Oliveira 3590 (7)

Oliveira 3885 (7)

Oliveira 4463 (7)

Paula 117 (SP)

Paula 70 (11)

Pedersen 14638 (4)

Philcox 4519 (11)

Pires 58041 (11)

Pittier 9154 (33)

Plowman 8267 (7)

Queiroz 1141 (34)

Queiroz 1240 (25)

Queiroz 1633 (8)

Ratter 4957 (21)

Ribeiro 37 (25)

Saldias 953 (28)

Scariot 474, 476 (13)

Scariot 482 (13)

Schinini 20288 (15)

Schinini 20291 (15)

Shamel 345b (11)

Smith 1460 (27)

Smith 6699 (29)

Steyermark 102432 (33)

Steyermark 110614 (33)

Swallen 9520 (6)

Tales 207 (11)

Taylor E1049 (7)

Toledo 43184 (21)

Tsuji 2622 (3)

Tsuji 2681 (13)

Tsuji 2706 (18)

Tsuji 974 (31)

Tsujiet 2703 (10)

Viera 178 (11)

Viera 201 (21)

Viera 262 (11)

Werdermann 2922 (8)

Werdermann 2988 (8)

Wurdack 40852 (27)

Zappi s.n (CFCR 9371; SPF 42629)(12)
